# The ESCAPE trial for older people with chronic low back pain: Protocol of a randomized controlled trial

**DOI:** 10.1371/journal.pone.0266613

**Published:** 2022-05-26

**Authors:** Hytalo de Jesus Silva, Leticia Soares Fonseca, Rodrigo Oliveira Mascarenhas, Júlio Pascoal de Miranda, Paulo André Almeida, Mateus Bastos Souza, Leani Souza Maximo Pereira, Murilo Xavier Oliveira, Vinicius Cunha Oliveira

**Affiliations:** 1 Postgradute Program in Rehabilitation and Functional Performance, Universidade Federal dos Vales do Jequitinhonha e Mucuri, Diamantina, Brazil; 2 Postgraduate Program in Health Sciences, Universidade Federal dos Vales do Jequitinhonha e Mucuri, Diamantina, Brazil; 3 Physical Therapy Department, Universidade Federal dos Vales do Jequitinhonha e Mucuri, Diamantina, Brazil; 4 Postgradute Program in Rehabilitation Sciences, Universidade Federal de Minas Gerais, Belo Horizonte, Brazil; Prince Sattam Bin Abdulaziz University, College of Applied Medical Sciences, SAUDI ARABIA

## Abstract

**Background:**

Low-back pain is one of the most common health conditions worldwide. It is defined as pain below the costal margin and above the inferior gluteal folds. Current guidelines recommend management of chronic health (e.g., low back pain) conditions in older people at primary health care settings using active strategies (e.g., exercise). In non-specific low back pain, high quality evidence supports active strategies for general population. However, the management of non-specific low back pain in the older people has been overlooked and evidence is limited to a small number of low powered randomized controlled trials with high risk of bias.

**Methods:**

This is a prospectively registered, open, two-arm randomised controlled trial comparing the group-based exercise and waiting list in pain intensity (11-item Pain Numerical Rating Scale) and disability (Roland Morris questionnaire) of older people (i.e., 60 years old or over) with chronic non-specific low back pain. One hundred and twenty patients will be recruited from Diamantina, Brazil. Follow-ups will be conducted in post-treatment (8 week) and 6- and 12-months post-randomisation.

**Discussion:**

Our hypothesis is that group-based exercise will be better than waiting list in reducing pain intensity and disability in older people with chronic non-specific low back pain.

**Impact:**

The practice of individualized exercise has been studied for the management of chronic non-specific low back pain in older people. However, the group exercise, even showing high quality evidence for the improvement of several important outcomes in this population, has been ignored until now. Thus, the results of this study have the potential to indicate a viable and accessible strategy for managing chronic non-specific low back pain in the older people.

**Trial registration:**

The study was prospectively registered at www.ensaiosclinicos.gov.br (RBR-9j5pqs). Date-11/18/2020.

## 1. Background

Low-back pain (LBP) is one of the most common health conditions worldwide [1]. It is defined as pain below the costal margin and above the inferior gluteal folds, with or without referred leg pain [2, 3]. It is expected that 70% to 85% of the general population will present an episode of LBP during lifetime, including older people (i.e., 60 years old or over) [4, 5]. The most common form of LBP is non-specific [6], defined as the symptoms without a specific cause [7, 8]. Older people with chronic non-specific (CNS) LBP reports disability [9, 10], productivity loss [11] and low quality of life (QOL) [12].

Current guidelines recommend management of chronic health conditions in older people at primary health care settings using active strategies (e.g., exercise) to decrease pain intensity, disability, productivity loss and health care costs [3, 13, 14]. In primary health care, group-based exercise is often used to manage older people [15, 16] because it has shown to be cost-effective for older people with different health conditions [17–19]. For instance, this active strategy is effective for prevention and fear of falling [17, 18], improvement of QOL [17] and balance [17, 20] in the older population.

In CNSLBP, high quality evidence supports active strategies such as exercise for general population [3, 14, 21–25]. Previous systematic reviews also showed promising efficacy of exercise on pain intensity and disability related to CNSLBP in older people [26]. However, the management of CNSLBP in this population has been overlooked and evidence is limited to a small number of low powered randomized controlled trials with high risk of bias [27, 28]. Moreover, efficacy of group-based exercise has not been investigated in older people with CNSLBP [26, 28]. Thus, the primary aim of this randomized controlled trials is to investigate the efficacy of an 8-week group-based exercise program on pain intensity and disability in older people with CNSLBP in a primary health care setting. Our secondary aims are to investigate efficacy on global impression of recovery, frequency of falls, fear of falling and physical activity (PA) level.

## 2. Methods

### 2.1 Elaboration protocol

**Fig 1:**
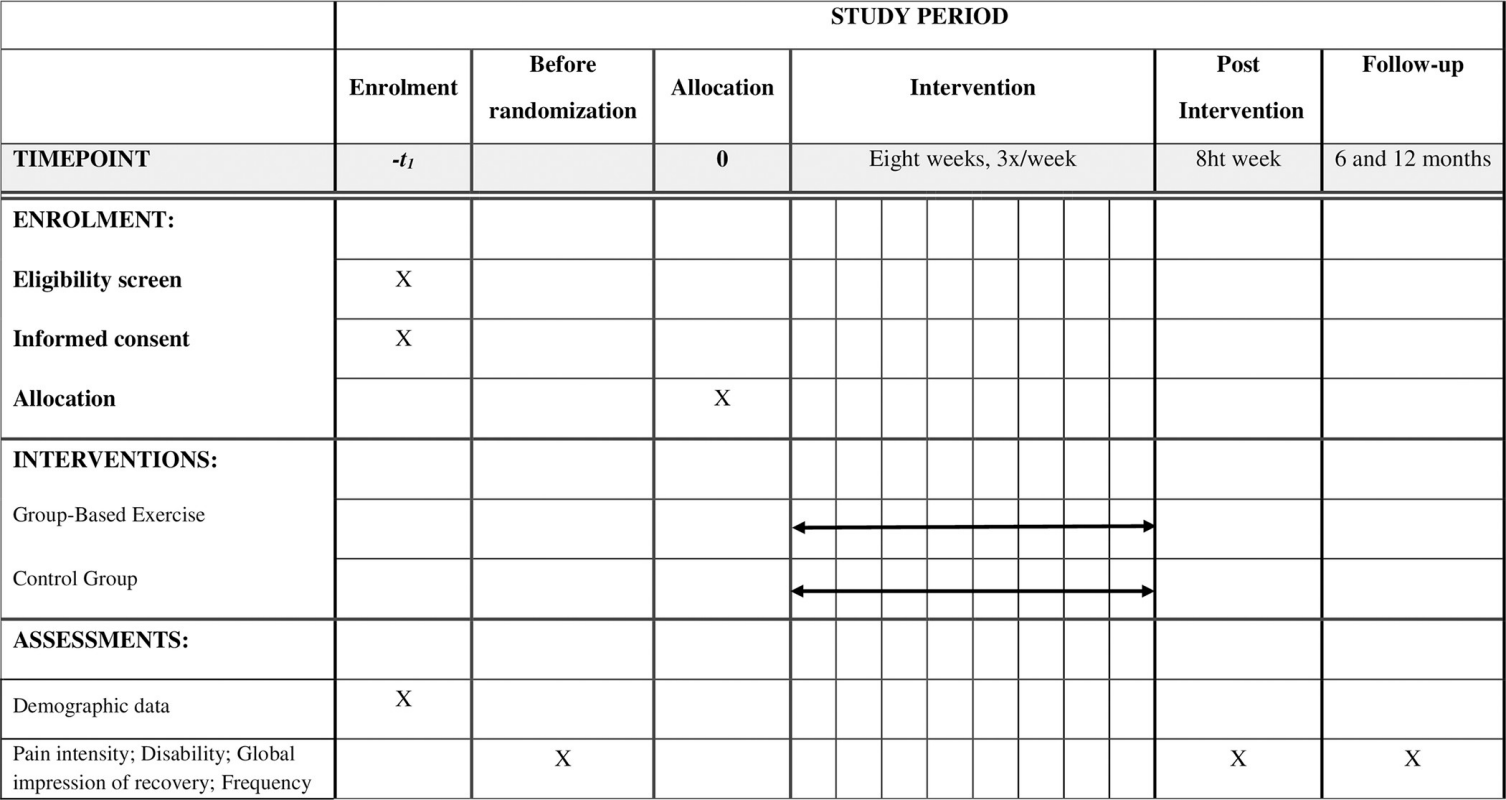
SPIRIT schedule of assessments at different time points.

This protocol was developed in accordance with the SPIRIT guidelines [29] and is reported according to the CONSORT statement [30]. The schedule with assessment at different points in time is shown in Fig 1.

### 2.2 Study design

**The Exercise for chronic back pain in older people (ESCAPE) trial** is a prospectively registered, open, two arm randomised controlled trial.

### 2.3 Settings and eligibility criteria

The study will be conducted at primary health care centers in Diamantina, Brazil. Participating in the study are patients aged 60 years old or over who seeks primary health care or a university outpatient physical therapy center in Diamantina, Brazil. The inclusion and exclusion criteria are as follows:

The inclusion criteria:

- Older people aged 60 years old or over who have CNSLBP, defined as pain below the costal margin and above the inferior gluteal folds, for at least 3 months [2, 3]; and
- Disability scores 4 out of 24 points or higher in the Roland-Morris Disability Questionnaire (RMDQ) and pain intensity scores 3 out of 10 points or higher in a 11-point numeric rating scale [31].

The exclusion criteria:

- Suspected or confirmed serious spinal pathology (fracture, metastatic, inflammatory or infective diseases of the spine, cauda equina syndrome/widespread neurological disorder);
- Radiculopathy (i.e., degree 2 of strength, reflex or sensation affected for same nerve root);
- Previous history of spinal surgery in the last 12 months; scheduled for major surgery during the study or at the follow up period;
- Indicative of cognitive deficit validated by the Mini Mental State Examination; and
- Contraindications to exercise listed of the American College of Sports Medicine (ACSM) guidelines (Table 1) [32].

**Table 1:** Contraindications to exercise testing.

**ABSOLUTE**
➢ A recent significant change in the resting electrocardiogram (ECG) suggesting significant ischemia, recent myocardial infarction (within 2 d), or other acute cardiac event ➢ Unstable angina ➢ Uncontrolled cardiac dysrhythmias causing symptoms or hemodynamic ➢ compromise ➢ Symptomatic severe aortic stenosis ➢ Uncontrolled symptomatic heart failure ➢ Acute pulmonary embolus or pulmonary infarction ➢ Acute myocarditis or pericarditis ➢ Suspected or known dissecting aneurysm ➢ Acute systemic infection, accompanied by fever, body aches, or swollen lymph glands
**RELATIVE (must consult a doctor before participation)**
➢ Left main coronary stenosis ➢ Moderate stenotic valvular heart disease ➢ Electrolyte abnormalities (*e*.*g*., hypokalemia or hypomagnesemia) ➢ Severe arterial hypertension (*i*.*e*., systolic blood pressure [SBP] of _200 mm Hg and/or a diastolic BP [DBP] of _110 mm Hg) at rest ➢ Tachydysrhythmia or bradydysrhythmia ➢ Hypertrophic cardiomyopathy and other forms of outflow tract obstruction ➢ Neuromotor, musculoskeletal, or rheumatoid disorders that are exacerbated by exercise ➢ High-degree atrioventricular block ➢ Ventricular aneurysm ➢ Uncontrolled metabolic disease (*e*.*g*., diabetes, thyrotoxicosis, or ➢ myxedema) ➢ Chronic infectious disease (*e*.*g*., HIV) ➢ Mental or physical impairment leading to inability to exercise adequately

Note: ECG = electrocardiogram; HIV = Human Immunodeficiency Virus; BP = blood pressure

### 2.4 Procedure and outcome measures

**Fig 2:**
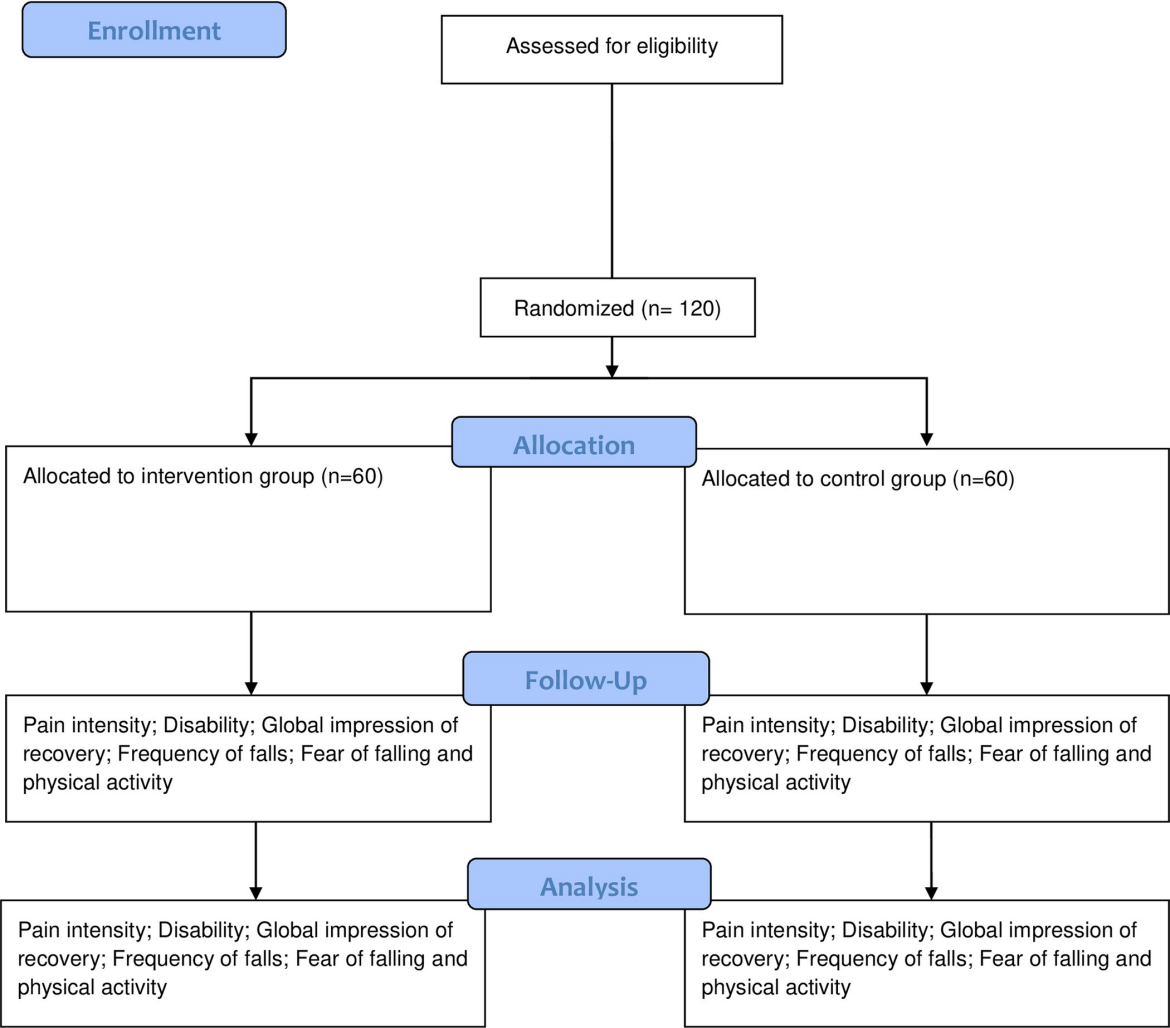
Flow diagram.

All eligible older people will receive information about the study and sign an informed consent form prior to participation. Baseline assessment (in person) will include age, sex, history of falls, duration of symptoms, prognosis rating (STarT Back Screening Tool) [33], functional capacity (Short Physical Performance Battery) [34], cognitive function (Mini Mental State Examination), and the primary and secondary outcomes. Primary and secondary outcomes will be assessed (in person) again at the end of the intervention (i.e., 8 weeks after randomization) and at the 6 and 12-months follow-up. Fig 2 represents the flow of participants during the study.

#### Primary outcomes

- Pain intensity over the previous week (measured with a 0–10 points in numeric rating scale [NRS]): The NRS is a self-reported scale in which the patient can select one number that best describes the pain intensity. The score ranges from 0 to 10; higher scores represent higher levels pain intensity. This scale was previously validated for population with low back pain [35, 36].
- Disability (measured with the 0–24 points in Roland-Morris Disability Questionnaire [RMDQ]): The RMDQ is a self-reported scale of 24 items in which the patient reports pain-related disability. The score per item ranges from 1 (yes) to 0 (no). Total score ranges from 0 to 24, higher scores represent higher levels of pain-related disability. This instrument was previously validated for the low back pain Brazilian population [37, 38].

#### Secondary outcomes

- Global impression of recovery (measured with the—3 to 3 Global Perceived Effect Scale [GPE]) [39]: GPE is a scale that assesses whether the patient condition has gotten worse, better, or stayed the same and to quantify the magnitude of that change. Total score ranges from—3 to 3 (i.e., -3 extremely worse, 0 unchanged and 3 fully recovered). This scale was previously validated for population with low back pain [40].
- Frequency of falls (number of falls during study time): All trial participants will be invited to self-complete a falls diary during study time. Falls diaries will be produced in a calendar format, printed in color on firm card. This cololette method was previously validated for the older people population [41].
- Fear of falling (measured with the 16–64 Falls Efficacy Scale–International [FES-I]) [42]: The FES-I presents questions about the concern with the possibility of falling when performing 16 activities, with respective scores from 1 to 4. The total score can range from 16 (i.e., no concern) to 64 (i.e., extreme concern). This instrument was previously validated for the older people Brazilian population [42].
- PA level (measured with the 1–7 Physical Activity Rating): Physical Activity Rating is a progressive scale with scores from 0 to 7, in which the most appropriate option for the history of physical activity in the last 30 days should be selected. Higher scores represent higher Physical Activity level. This instrument was previously validated for the older people Brazilian population [43].

### 2.5 Randomisation

The randomisation sequence to our two groups of interest (i.e., group-based exercise or control) will be computer-generated by one of the investigators who will not be involved in the recruitment of participants. The sequence will be blocked (block sizes of 4, 6, and 8, in random order). Allocation will be concealed in sequentially numbered, sealed, opaque envelopes. Participants will be stratified by gender (female or male).

### 2.6 Blinding

The statistician will be blinded to treatment allocation. The data will be coded in an unidentifiable manner and will not contain any information that can reveal what group a single participant or a group were randomised to. As this is an exercise program, it will not be possible to blind the therapist and the patient. Also, because our outcomes are Patient‐Reported Outcome Measures (PROMs), it will not be possible to blind the assessor. However, PROMs are validated and widely accepted in research and the clinic.

### 2.7 Intervention

#### Group-based exercise

The group-based exercise (GBE) programme will be delivered by two physical therapists (experience in musculoskeletal rehabilitation in primary health care). The GBE comprises three sessions per week of group-based exercise in a local community center, for 8 weeks [44]. Each group session will consist of 10 to 18 participants and each exercise session will last 60 minutes and consist of four stages: (1) five minutes warm up (i.e., self-regulated walk); (2) twenty minutes of moderate intensity walking; (3) thirty minutes of resistance training for the major muscles of the leg, trunk and arm and balance exercises that progress in difficulty; and (4) five-minute cool down period (i.e., self-regulated walk). The exercise intensity will be assessed by one of the following criteria: (1) point of volitional fatigue [45] for the resistance training component; and (2) perceived effort ranging from 3 to 4 on the modified 10-point Borg scale score for the walking component [45]. The intensity will be increased along the 8-week programme following the recommendations of the ACSM guideline [32] (i.e., resistance exercise- a gradual progression of more repetitions per set; and aerobic exercise- a gradual progression of exercise volume by adjusting exercise intensity) [32].

The GBE programme was set up based on tasks that makes part of performance-based assessment instruments widely used for the evaluation of physical performance of the older people (i.e., Berg Balance Scale [46], Dynamic Gait Index [47] and Timed Up and Go [48]). In addition, exercises commonly used in primary health care groups were added [49–51]. The protocol will take place in a gymnasium and will be supervised by a trained physical therapist [52–54]. Examples of the choices of exercises are provided in the S1 Appendix.

Modifications were performed after registration of the clinical trial. We included 5 minutes of warm-up and 5 minutes of cool-down during the intervention. To reduce the risk of injury during the protocol. Furthermore, based on recommendations of the ACSM guideline, criteria were established to intensity increase along the 8-week. All these changes were made before starting to recruit participants. Furthermore, the registry has been updated and is available with new information.

#### Control group

Participants randomly allocated to control group will remain on a waiting list. In addition, weekly contact will be made to ensure that they do not start treatment during the study protocol. However, previous treatments like medications will be allowed.

### 2.8 Data analysis

#### Sample size calculation

The sample size calculation was performed using the G*Power 3.1 software. The difference between means of a meta-analysis 1.7 [26] was divided by the standard deviation of the study of Zadro (2019) [55] 4.80 to obtain Cohen’s d. Considering the Cohen’s d, a sample size of 120 participants was calculated (60 in each group), with a statistical power of 80%, alpha of 5%, and 20% dropout rate.

#### Analysis of treatment effects

The statistical analysis will be performed following the intention-to-treat analysis principles [56, 57]. The blinded statistician will be given coded data, the normality will be tested using the Kolmogorov-Smirnov test and the homoscedasticity of the data will be tested using the Levene test. Then, considering normal distribution, an analysis of mixed linear models (random intercepts and fixed coefficients) will be conducted, which incorporated terms for treatment, time, and the treatment-time interactions. As two physical therapists will be responsible for implementing the GBE will be applied multilevel analyses with participants nested by physiotherapist. In this sense, level 1 being the different time points, level 2 the subject, and level 3 the physical therapists.

Mean differences and their confidence intervals (CIs) at 95% will be presented. Primary outcome results will be interpreted based on the minimal clinically important difference (MCID) estimated for the adult population (i.e., RMDQ > 5 and NRS > 2) points [58]. All statistical analyses will be performed with SPSS (IBM Corp. Released 2013. IBM SPSS Statistics for Windows, Version 22.0. Armonk, NY: IBM Corp).

In addition, the sample will be dichotomized for improvement / maintenance according to the clinically important differences. The absolute risk reduction will be obtained by subtracting the GBE group risk by the control group risk. This data will be used to calculate the number needed to treat (NNT) (100% / by reducing the absolute risk).

### 2.9 Implementation and study group

The project will involve primary care centers that care for older peoples with CNSLBP or a university outpatient physical therapy center in Diamantina, Brazil. Older people referred to treat the condition of interest in the included settings will be invited. If there is an interest in participating, they will undergo an eligibility screening with a physiotherapist. We hypothesise that the GBE program will benefit older peoples with CNSLBP by reducing disability, pain intensity, global impression of recovery, frequency of falls, fear of falling and PA level compared with waiting list.

### 2.10 Plan for supervision and monitoring

The study will be conducted and monitored by the lead investigator (HJS) under the supervision of the coauthor (VCO), with assistance of the research team. All the ethical principles as provided by Declaration of Helsinki will be followed by all the members of this research throughout the study.

### 2.11 Plan for data integrity and management

The research data will be collected by a research assistant who will be trained to collect and manage it. Participant identifiers (including name, address and contact information) will be removed from the research data and will be stored separately. Data will be entered in Microsoft Excel. Research data will be monitored weekly by scrutinising entered data. Any errors in entry will be identified (if any) and amended. Consent forms will be scanned and stored in password-protected computers of the lead researcher and at the University’s along with other research data files.

### 2.12 Ethics

Participants will be informed about the study and will sign an informed consent form before participating in the trial. This study was approved by the Universidade Federal dos Vales do Jequitinhonha e Mucuri (UFVJM) Ethics Committee (number 37088920.5.0000.5108) on October 20, 2020. The protocol was prospectively registered at www.ensaiosclinicos.gov.br (RBR-9j5pqs). Protocol modifications will be reported to the Institutional Review Board and to the trial registry.

## 3. Discussion

### 3.1 Potential study impact and significance

CNSLBP results in a high rate of disability [9, 10] and in high direct and indirect healthcare costs for both patients and healthcare systems [59]. Among direct costs, a predominance of spending on physiotherapy (17%) and inpatient services (17%), followed by pharmacy (13%) and primary care (13%), were observed. As indirect costs, productivity loss predominates [59].

The findings of this study protocol may be useful for physio therapist and their patients, as they could have the potential to identify a viable and accessible strategy for CNSLBP management in older people. From the point of view of physio therapist the group based exercised is a practical intervention, inexpensive and applicable to clinical practice in different levels of healthcare [60–63]. On the other hand, patients can benefit from an easy-to-access and low-cost intervention, which has already shown efficacy in different outcomes in this population [17, 18, 20].

### 3.2 Study strengths and weaknesses

The main strength of this study protocol is that it is a randomized controlled trial with concealed allocations. The sample size was calculated to provide adequate statistical power to detect intergroup differences in the primary outcome. The physio therapist responsible for supervising the strengthening protocol will have similar clinical experience and will receive prior training.

The main limitation of our study is that the participants and therapists will not be blinded to the group allocations. In addition, it is not possible to affirm the blinding of the evaluator due to the fact that our outcomes are reported by patients, so the patient is his own evaluator.

### 3.3 Future research

The results of this study protocol could contribute to future studies comparing the effect of group exercise with different interventions used to manage CNSLBP in the older people.

## Supporting information

S1 Appendix Group exercise protocol. (DOCX)

S2 Appendix SPIRIT 2013 checklist. (DOC)

S3 Appendix TIDieR-checklist. (DOCX)

S1 File (DOCX)

S2 File (DOCX)

## List of abbreviations

LBP: Low back pain
QOL: quality of life
PA: physical activity
GBE: group-based exercise
RMDQ: Roland-Morris Disability Questionnaire
ACSM: American College of Sports Medicine
NRS: numeric rating scale
GPE: Global Perceived Effect Scale
MCID: Minimal clinically important difference
