# Supplementary material for: The ESCAPE trial for older people with chronic low back pain: Protocol of a randomized controlled trial
Source: PLoS One. 2022 May 26;17(5):e0266613. doi: 10.1371/journal.pone.0266613 (PMC9135264; doi:10.1371/journal.pone.0266613)
Supplement: S1 Appendix — (DOCX) [file pone.0266613.s001.docx]

| **Appendix S1 - Group Exercise Protocol**  **Balance Training** | | |
| --- | --- | --- |
| **TASK** | **DESCRIPTION** | **PRESCRIPTION** |
| **3º and 4º weeks** | | |
| Reach ahead | Lift your arm 90 degrees. Stretch your fingers and try to reach ahead as far as possible | Self-regulation |
| Step over obstacle | Begin walking at your normal speed. When you come to the shoe box, step over it, not around it, and keep walking. | 10 meters walk with 5 boxes |
| Rotate 360º | Rotate completely around yourself. Break. Rotate completely around yourself to the opposite side. | 3 sets of 5 repetitions |
| Timed up and go – modified | Sit on a chair, get up and walk forward to a mark on the floor, turn back and sit on the chair | 3 sets of 12 repetitions |
| **5º and 6º weeks** | | |
| Gait with horizontal head turns | Begin walking at your normal pace. When I tell you to “look right”, keep walking straight, but turn your head to the right. Keep looking to the right until I tell you, “look left”, then keep walking straight and turn your head to the left. Keep your head to the left until I tell you, “look straight,” then keep walking straight, but return your head to the center | 10 meters walk with 5 double task points |
| Rotate 360º | Rotate completely around yourself. Break. Rotate completely around yourself to the opposite side. | 3 sets of 5 repetitions |
| Gait witch vertical head turns | Begin walking at your normal pace. When I tell you to “look up” keep walking straight, but tip your head and look up. Keep looking up until I tell you,” look down”. Then keep walking straight and turn your head down. Keep looking down until I tell you “look straight” then keep walking straight, but return your head to the center | 10 meters walk with 5 double task points |
| Timed up and go – modified | Sit on a chair, get up and walk forward to a mark on the floor, turn back and sit on the chair | 3 sets of 12 repetitions |
| Tandem position | Place one foot directly in front of the other on the same line; if you think you will not be successful, place your foot a little further in front of the other foot and slightly to the side. | 3 sets of 12 repetitions |
| **7ª and 8ª weeks** | | |
| Gait with horizontal head turns | Begin walking at your normal pace. When I tell you to “look right”, keep walking straight, but turn your head to the right. Keep looking to the right until I tell you, “look left”, then keep walking straight and turn your head to the left. Keep your head to the left until I tell you, “look straight,” then keep walking straight, but return your head to the center | 10 meters walk with 5 double task points |
| Gait witch vertical head turns | Begin walking at your normal pace. When I tell you to “look up” keep walking straight, but tip your head and look up. Keep looking up until I tell you,” look down”. Then keep walking straight and turn your head down. Keep looking down until I tell you “look straight” then keep walking straight, but return your head to the center | 10 meters walk with 5 double task points |
| Step over obstacle | Begin walking at your normal speed. When you come to the shoe box, step over it, not around it, and keep walking. | 10 meters walk with 5 boxes |
| Walking Heel to Toe | Place your right foot in front of your left foot so that the heel of your right foot touches the top of your left toes. Move your left foot in front of your right, putting the weight on your heel. Then shift your weight to your toes. Repeat the step with your left foot. | 5 meters |

| **STRENGTHENING** | | | |
| --- | --- | --- | --- |
| **1º and 2º weeks** | | |  |
| **Squat** | Raise from a chair sit to stand | 3 sets of 12 repetitions | |
| **Hip abduction** | With a extended knee, lift your leg out to the side. Slowly lower your leg so your foot is back on the floor. | 3 sets of 12 repetitions. | |
| ***Hip extension*** | In prone position, raise leg | 3 sets of 12 repetitions. | |
| ***Abdominals from lying position*** | Slide the heel towards the gluteal region activating the lower abdominals | 3 sets of 12 repetitions. | |
| ***Bridge exercise*** | In ventral decubitus, with his knees flexed and his feet resting on the ground. Keep your arms next to your body with your palms down. Lift your hips off the ground until your knees, hips and shoulders form a straight line. | 3 sets of 12 repetitions. | |
| ***Isometric Board*** | A front plank should be performed keeping the body in a straight line with the elbows and feet flat on the floor. |  | |
| ***Step up*** | Climb the step first with the right leg, then with the left. | 3 sets of 12 repetitions. | |
| ***Strengthening of sural triceps*** | Getting up, lift your heel off the floor and lower again. | 3 sets of 12 repetitions. | |
| ***Push-ups against the wall*** | Stand your body forward and rest your hands on the wall. Start the movement of flexing and extending arms. | 3 sets of 12 repetitions. | |
| **3º and 4º weeks** | | |  |
| ***Bridge Exercise*** | In ventral decubitus, with his knees flexed and his feet resting on the ground. Keep your arms next to your body with your palms down. Lift your hips off the ground until your knees, hips and shoulders form a straight line. | 3 sets of 12 repetitions. | |
| ***Isometric Board*** | A front plank should be performed keeping the body in a straight line with the elbows and feet flat on the floor. |  | |
| ***Back Extensors*** | In the supine position, as the body declines, a pillow under the stomach and arms at the side of the body, lift the torso to the neutral position. | 3 sets of 12 repetitions. | |
| **Squat** | Raise from a chair sit to stand | 3 sets of 12 repetitions | |
| ***Step up*** | Climb the step first with the right leg, then with the left. | 3 sets of 12 repetitions. | |
| ***Push-ups Against wall*** | Stand your body forward and rest your hands on the wall. Start the movement of flexing and extending arms. | 3 sets of 12 repetitions. | |
| **5º and 6º weeks** | | |  |
| ***Bridge exercise with leg extension*** | In ventral decubitus, with his knees flexed and his feet resting on the ground. Keep your arms next to your body with your palms down. Lift your hips off the ground until your knees, hips and shoulders form a straight line. Lift leg in extension. | 3 sets of 12 repetitions. | |
| ***Lower abdominal cycling*** | Raise your legs straight towards the ceiling, and perform the pedaling movement while raising the trunk. | 3 sets of 12 repetitions. | |
| ***Jumping jack*** | Raise your arms while you make the movement to open and close both legs. | 3 sets of 12 repetitions. | |
| ***Oblique*** | In lateral decubitus supported on the knees and elbow, perform hip elevation. | 3 sets of 12 repetitions. | |
|  | Alternating arm / leg extensions from the position of knees of four support. | 3 sets of 12 repetitions. | |
| ***Step up*** | Climb the step first with the right leg, then with the left. | 3 sets of 12 repetitions. | |
| ***Press-ups with Modified knee flexion*** | In the plank position with support on your hands and knees, perform the arm flexion movement. | 3 sets of 12 repetitions. | |
| **7º and 8º weeks** | | |  |
| ***Bridge exercise with leg extension*** | In ventral decubitus, with his knees flexed and his feet resting on the ground. Keep your arms next to your body with your palms down. Lift your hips off the ground until your knees, hips and shoulders form a straight line. Lift leg in extension. | 3 sets of 12 repetitions. | |
| ***Lower abdominal cycling*** | Raise your legs straight towards the ceiling, and perform the pedaling movement while raising the trunk. | 3 sets of 12 repetitions. | |
| ***Abdominal strengthening exercises for oblique abdominal muscles*** | With your knees bent, raise and lateralize the upper trunk. | 3 sets of 12 repetitions. | |
| ***Jumping jack*** | Raise your arms while you make the movement to open and close both legs. | 3 sets of 12 repetitions. | |
| ***Oblique*** | In lateral decubitus supported on the knees and elbow, perform hip elevation. | 3 sets of 12 repetitions. | |
|  | Alternating arm / leg extensions from the position of knees of four support. | 3 sets of 12 repetitions. | |
| ***Press-ups with Modified knee flexion*** | In the plank position with support on your hands and knees, perform the arm flexion movement. | 3 sets of 12 repetitions. | |

| **AEROBIC** | | |  |
| --- | --- | --- | --- |
| **1º - 8º weeks** | | |  |
| **TASK** | **DESCRIPTION** | **PRESCRIPTION** | |
| ***Walking*** | Self-regulated walk. | 20 minutes | |
