# Supplementary material for: The ESCAPE trial for older people with chronic low back pain: Protocol of a randomized controlled trial
Source: PLoS One. 2022 May 26;17(5):e0266613. doi: 10.1371/journal.pone.0266613 (PMC9135264; doi:10.1371/journal.pone.0266613)
Supplement: S1 File — (DOCX) [file pone.0266613.s004.docx]

**PARECER CONSUBSTANCIADO DO CEP**

# DADOS DO PROJETO DE PESQUISA

**Título da Pesquisa:** Eficácia do exercício na dor e incapacidade relacionada à dor lombar crônica em idosos: Um ensaio clínico randomizado

**Pesquisador:** VINICIUS CUNHA DE OLIVEIRA

# Área Temática:

**Versão:** 3

**CAAE:** 37088920.5.0000.5108

**Instituição Proponente:** Universidade Federal dos Vales do Jequitinhonha e Mucuri

**Patrocinador Principal:** Financiamento Próprio

# DADOS DO PARECER

**Número do Parecer:** 4.350.616

# Apresentação do Projeto:

“As informações aqui elencadas foram retiradas do arquivo Informações Básicas da Pesquisa (PB_Informações_Básicas_do_projeto_1563109.pdf, de 18/10/2020).

A dor lombar é uma das condições de saúde mais comuns em todo o mundo. Estíma-se que 70% a 85% da população geral apresentou ou apresentará um episódio de dor lombar durante a vida, incluindo idosos. A dor lombar mais comum é a inespecífica, definida como os sintomas sem uma causa específica. Evidencias de alta qualidade apoiam estratégias ativas, como exercícios para a população para tratamento de dor lombar inespecífica na gera. Uma revisão anterior realizada pelo nosso grupo de pesquisa também mostrou eficácia promissora do exercício sobre dor e incapacidade relacionada à dor lombar em idosos. No entanto, o manejo da lombalgia inespecífica na população idosa foi negligenciada na literatura científica, por isso as evidências são fracas e limitadas a um pequeno número de ensaios clínicos randomizados de baixa potência e alto risco de viés. Além disso, a eficácia do exercício em grupo não foi investigada em idosos com dor lombar inespecífica. OBJETIVO - Investigar o efeito de um protocolo de 8 semanas de exercício em grupo sobre a dor e incapacidade em idosos com dor lombar inespecífica. MÉTODOS – Idosos com 60 anos ou mais serão avaliados no período pré e pós intervenção, alem do acompanhamento de 6 e 12 meses. Os questionários que serão aplicados são os seguintes: Escala Numérica de Dor (0 - 10); Questionário de Roland-Morris (0 - 24); Escala de percepção do efeito global (-5 - 5); Falls

Efficacy Scale – International (16-64); Physical Activity Rating (0 -7). Após a avaliação pré intervenção os participantes serão aleatoriamente distribuídos entre dois grupos. Grupo controle/Grupo de exercício em grupo. Os exercícios em grupo serão realizados 3 vezes por semana durante 8 semanas e o grupo controle permanecerá na lista de espera. A alocação dos participantes para cada grupo será realizada utilizando envelope opaco.

- Hipótese nula – O protocolo de exercício em grupo não diferente do grupo controle para dor e incapacidade em idosos com dor lombar.
- Hipótese alternativa – O protocolo de exercício em grupo melhora a dor e a incapacidade de idosos com dor lombar. Esses efeitos são mantidos após 6 e 12 meses de acompanhamento. Além disso idosos que particparam do grupo apresentarão maior nível de atividade física após 12 meses de acompanhamento.

Critério de Inclusão:

·Idosos de ambos os sexos com 60 anos ou mais;

·Queixa de dor lombar inespecífica com pelo menos 3 meses de duração;

·Incapacidade com pontuação 4/24 ou mais no RMDQ nos últimos seis meses e dor nas costas persistente com dor 3/10 ou mais nos últimos seis meses (RUNDELL et al., 2017).

Critério de Exclusão:

•Suspeita ou confirmação de patologia espinhal grave (fratura, doenças metastáticas, inflamatórias ou infecciosas da coluna vertebral, síndrome da cauda equina / distúrbio neurológico generalizado);

-Compromisso da raiz nervosa;

-Cirurgia da coluna vertebral;

-Grandes cirurgias agendadas durante o período de tratamento ou acompanhamento;

-Qualquer uma das contra-indicações para o exercício listadas na pagina 103 da diretrizes do ACSM (DALLECK; TISCHENDORF, 2012) (Anexo 1)

# Objetivo da Pesquisa:

“As informações aqui elencadas foram retiradas do arquivo Informações Básicas da Pesquisa (PB_Informações_Básicas_do_projeto_1563109.pdf, de 18/10/2020).

Objetivo Primário:

Investigar o efeito de um protocolo de 8 semanas de exercício em grupo sobre a dor e incapacidade em idosos com dor lombar inespecífica.

Objetivos Secundários:

- Investigar o efeito de um protocolo de 8 semanas de exercício em grupo sobre a impressão global de recuperação de idosos com dor lombar inespecífica.
- Investigar o efeito de um protocolo de 8 semanas de exercício em grupo sobre o medo de cair de idosos com dor lombar inespecífica.
- Investigar o efeito de um protocolo de 8 semanas de exercício em grupo sobre a frequência de quedas em idosos com dor lombar inespecífica.
- Investigar o efeito de um protocolo de 8 semanas de exercício em grupo sobre o nível de atividade física em idosos com dor lombar inespecífica após 12 meses de acompanhamento.

# Avaliação dos Riscos e Benefícios:

“As informações aqui elencadas foram retiradas do arquivo Informações Básicas da Pesquisa (PB_Informações_Básicas_do_projeto_1563109.pdf, de 18/10/2020).

Riscos:

Os riscos para participação no presente estudo são mínimos. OS PARTICIPANTES DO GRUPO INTERVENÇÃO poderão apresentar dor muscular de baixa intensidade após a realização do programa de exercícios. Caso ocorra, para minimizar a dor, será realizada termoterapia pelo pesquisador e será orientado alongamento em leve escala. PELO FATO DO GRUPO CONTROLE PERMANECER EM LISTA DE ESPERA (NÃO REALIZAR QUALQUER INTERVENÇÃO) OS RISCOS SÃO AINDA MENORES. UM POTENCIAL RISCO AOS PARTICIPANTES DESTE GRUPO É O FATO QUE ELES SERÃO ORIENTADOS A NÃO INICIAR QUALQUER TRATAMENTO DURANTE O PROTOCOLO DO ESTUDO, DESSA FORMA, O MESMO PASSARÁ 8 SEMANAS SEM UMA NOVA INTERVENÇÃO PARA TRATAMENTO DE SUA DISFUNÇÃO. COMO FORMA DE MINIMIZAR, OS PARTICIPANTES SERÃO INFORMADOS QUE ISSO SETRATA DE UMA ORIENTAÇÃO, CASO QUEIRAM INICIAR UM NOVO TRATAMENTO DURANTE O ESTUDO ELES NÃO SERÃO IMPEDIDOS. ALÉM DISSO, NÃO SERÁ SOLICITADA A INTERRUPÇÃO DE TRATAMENTOS JÁ REALIZADOS. OS PARTICIPANTES TAMBEM SERÃO ORIENTADOS A CONTACTAR OS PESQUIDADORES EM CASO DE PIORA DO QUADRO CLÍNICO, CASO OCORRA SERÁ REALIZADA UMA AVALIAÇÃO PARA IDENTIFICAÇÃO DE POSSÍVEIS CAUSAS E SERÁ FORNECIDO AO PARTICIPANTE INTERVENÇÕES PARA CORREÇÃO DO QUADRO CLÍNICO, COMO POR EXEMPLO TERMOTERAPIA E ALONGAMENTO EM LEVE ESCALA PARA ANALGESIA.

Benefícios:

A participação no presente estudo trará benefícios diretos e indiretos. Os benefícios diretos serão através de melhoras em diferentes domínios da saúde causada pela atividade física. Como benefícios indiretos os resultados desta pesquisa poderão contribuir para auxiliar o profissional de fisioterapia na implementação de protocolos de tratamento eficazes e financeiramente viável para pacientes idosos com dor lombar.

# Comentários e Considerações sobre a Pesquisa:

“As informações aqui elencadas foram retiradas do arquivo Informações Básicas da Pesquisa (PB_Informações_Básicas_do_projeto_1563109.pdf, de 18/10/2020).

Metodologia de análise de dados:

Cálculo do tamanho da amostra: O cálculo do tamanho da amostra foi realizado usando o software G*Power

3.1. A diferença entre as médias de uma metanálise realizada por nosso grupo de pesquisa (dados a serem publicados) foi dividida pelo desvio padrão do estudo de ZADRO (2019) para obter o d de Cohen. Os dados d de Cohen foram usados para gerar o tamanho do efeito f. São necessários 120 participantes (60 em cada grupo), considerando um poder estatístico de 80%, alfa de 5% e taxa de abandono de 20%.

Análise dos efeitos do tratamento.

A análise estatística será realizada seguindo os princípios de análise de intenção de tratar por meio do método de impultação múltipla (MCCOY, 2017). Primeiro, a normalidade dos dados será testada pelo teste de Kolmogorov-Smirnov e a homocedasticidade dos dados será testada pelo teste de Levene. Então, considerando a distribuição normal, a análise de variância multivariada de medidas repetidas (MANOVA) será usada para determinar os efeitos de 12 semanas de intervenção e 6 e 12 meses de acompanhamento. Diferenças significativas detectadas na análise de variância serão examinadas novamente com uma análise post hoc de LSD com o nível alfa corrigido de 0,05 para múltiplas comparações. O tamanho do efeito será calculado e definido como pequeno (0,2), médio (0,5) e grande (0,8). (COHEN, [s.d.]) Os desfechos primários serão analisados com base na medida clinicamente importante para população idosa que será desenvolvida durante o estudo. Todas as análises estatísticas serão realizadas com SPSS versão 22, e os resultados serão apresentados como médias e 95% de confiança intervalos (ICs). A amostra será dicotomizada para melhoria / manutenção de acordo com a mudança clinicamente importante para adultos.

A redução do risco absoluto será obtida subtraindo o risco do GI pelo risco do GC. Esses dados serão usados para calcular o número necessário para tratar (NNT) (100% / reduzindo o risco absoluto). Dicotomizaremos nossa amostra em dois grupos com base nas pontuações GPE. Será considerado como melhora quando os participantes pontuarem 3 (completamente recuperados) ou 2 (muito recuperados) e estáveis quando pontuarem 1 (ligeiramente recuperados) e 0 (sem alteração) ou -1 (ligeiramente pio). Para determinar a mudança clinicamente importante (MCI) do QIRM e END utilizaremos uma curva Receiver Operating Characteristic (ROC) (DE VET et al., 2007) (PORTNEY; WATKINS, 2008). O ponto de corte da curva ROC será calculado identificando o ponto na curva mais próximo do canto superior esquerdo, considerado o melhor corte para o qual a soma das percentagens de classificações de falsos positivos e falsos negativos ([1 - sensibilidade] + [1 - especificidade]) é o menor (DE VET et al., 2007). A responsividade será avaliada examinando as áreas sob a curva ROC (AUC) e as correlações entre os escores de mudança de QIRM e END e GPE. A AUC será obtida para descrever a capacidade do QIRM e do END de distinguir participantes que apresentaram melhora de participantes estáveis (PORTNEY; WATKINS, 2008). AUC de 0,50 indica que o questionário não tem acurácia diagnóstica além do acaso, enquanto um valor de 1,00 indica acurácia perfeita (PORTNEY; WATKINS, 2008). AUC de pelo menos 0,70 foi considerada adequada (TERWEE et al., 2007).

Previsão de início do trabalho: 01/02/2021 Previsão de finalização do Trabalho: 01/02/2024

# Considerações sobre os Termos de apresentação obrigatória:

Vide campo:“Conclusões e Pendências e Lista de Inadequações”

# Recomendações:

Vide campo:“Conclusões e Pendências e Lista de Inadequações”

# Conclusões ou Pendências e Lista de Inadequações:

“As informações aqui elencadas foram retiradas do arquivo Informações Básicas da Pesquisa (PB_Informações_Básicas_do_projeto_1563109.pdf, de 18/10/2020).

Pendência 1: “Riscos e Beneficios - não incluem os riscos do Grupo Controle e nem quais as medidas serão tomadas para minimiza-los. O voluntário, mesmo no grupo controle, é responsabilidade do pesquisador, pois ele é parte da pesquisa.”

Resposta à pendência 1: O tópico “Riscos e Benefícios” foi alterado para atender a solicitação, desta forma foi inserida a seguinte informação: Os riscos para participação no presente estudo são

mínimos. OS PARTICIPANTES DO GRUPO INTERVENÇÃO poderão apresentar dor muscular de baixa intensidade após a realização do programa de exercícios. Caso ocorra, para minimizar a dor, será realizada termoterapia pelo pesquisador e será orientado alongamento em leve escala. PELO FATO DO GRUPO CONTROLE PERMANECER EM LISTA DE ESPERA (NÃO REALIZAR QUALQUER INTERVENÇÃO) OS RISCOS SÃO AINDA MENORES. UM POTENCIAL RISCO AOS PARTICIPANTES DESTE GRUPO É O FATO QUE ELES SERÃO ORIENTADOS A NÃO INICIAR QUALQUER TRATAMENTO DURANTE O PROTOCOLO DO ESTUDO, DESSA FORMA, O MESMO PASSARÁ 8 SEMANAS SEM UMA NOVA INTERVENÇÃO PARA TRATAMENTO DE SUA DISFUNÇÃO. COMO FORMA DE MINIMIZAR, OS PARTICIPANTES SERÃO INFORMADOS QUE ISSO SETRATA DE UMA ORIENTAÇÃO, CASO QUEIRAM INICIAR UM NOVO TRATAMENTO DURANTE O ESTUDO ELES NÃO SERÃO IMPEDIDOS. ALÉM DISSO, NÃO SERÁ SOLICITADA A INTERRUPÇÃO DE TRATAMENTOS JÁ REALIZADOS. OS PARTICIPANTES TAMBEM SERÃO ORIENTADOS A CONTACTAR OS PESQUIDADORES EM CASO DE PIORA DO QUADRO CLÍNICO, CASO OCORRA SERÁ REALIZADA UMA AVALIAÇÃO PARA IDENTIFICAÇÃO DE POSSÍVEIS CAUSAS E SERÁ FORNECIDO AO PARTICIPANTE INTERVENÇÕES PARA CORREÇÃO DO QUADRO CLÍNICO, COMO POR EXEMPLO TERMOTERAPIA E ALONGAMENTO EM LEVE ESCALA PARA ANALGESIA.

Pendência 2: “Carta de co-partícipe de Senador Modestino Gonçalves - esta deverá ser reelaborada, pois

não é uma carta de anuência e sim de Instituição Co-partícipe. Esta deverá ser datada após esta análise inicial deste CEP. Assim já pode ser acrescentada na nova submissão.”

Resposta à pendência 2: A carta de co-partícipe de Senador Modestino Gonçalves foi inserida como anexo na plataforma na presente submissão.

Pendência 3 “TCLE - é necessário descrever quais os exercícios serão realizados pelos indivíduos do grupo intervenção (os participantes necessitam ter informação dos exercícios realizados). Além disso, como dito anteriormente, é necessário descrever os riscos do grupo controle e a forma de minimizá-los.

Resposta à pendência 3: As descrições dos exercícios foram inseridas no TCLE como solicitado da seguinte forma:

“OS EXERCÍCIOS QUE SERÃO REALIZADOS SÃO FREQUENTIMENTE UTLIZADOS EM PROGRAMAS DE EXERCÍCIO EM GRUPO E SERÃO OS SEGUINTES:

1. FORTALECIMENTO DAS PERNAS - SERÃO REALIZADOS EXERCÍCIOS COMO SENTAR E LEVANTAR DE UMA CADEIRA, AGACHAMENTO COM UMA PERNA NA FRENTE DA OUTRA, FICAR NA PONTA DOS PÉS, ABRIR E FECHAR AS PERNAS DEITADO DE LADO, LEVANTAR E ABAIXAR A PERNA DEITADO DE

BARRIGA PARA BAIXO, SUBIDA E DESCIDA DE DEGRAUS.

1. FORTALECIMENTO DO TRONCO - SERÃO REALIZADOS EXERCÍCIOS COMO, EXERCÍCIO DE PONTE, PRANCHA MANTENDO O CORPO RETO APOIADO NO COTOVELO E NOS PÉS, FORTALECIMENTO DE ABDOMINAIS, MOVIEMNTO DE PEDALAR BICICLETA DEITO DE BARRIGA PARA CIMA
2. FORTALECIMENTO DOS BRAÇOS – SERÃO REALIZADOS EXERCÍCIOS COMO, LEVANTAR OS BRAÇOS ATÉ A ALTURA DOS OMBROS, FAZ O MOVIMENTO DE FLEXÃO NA PAREDE E MOVIMENTO DE FLEXÃO APOIADO NO JOELHO.
3. EXERCÍCIOS DE EQUILÍBRIO COMO, FICAR PARADO EM UMA PERNA SÓ, CAMINHAR ENCOSTANDO A CALCARNHA DE UM PÉ NA PONTA DO OUTRO, DESVIAR DE CONES DE CONES, PASSAR POR CIMA DE CAIXAS DE SAPATO.
4. CAMINHADA DE 10 MINUTOS.”

Os riscos para o grupo controle foram descritos no TCLE com solicitado da seguinte forma:

“CASO VOCÊ SEJA SORTEADO PARA O GRUPO CONTROLE, PELO FATO DE PERMANECER EM LISTA DE ESPERA (NÃO REALIZAR QUALQUER TRATAMENTO) OS RISCOS SÃO AINDA MENORES. UM RISCO QUE PODE SER CITADO É O FATO QUE VOCÊ SERÁ ORIENTADO A NÃO INICIAR QUALQUER TRATAMENTO DURANTE O PROTOCOLO DO ESTUDO, DESSA FORMA PASSARÁ 8 SEMANAS SEM UM NOVO TRATAMENTO PARA SUA DOR NAS COSTAS. COMO FORMA DE MINIMIZAR ESSE RISCO, DEIXAMOS CLARO

QUE ISSO SE TRATA DE UMA ORIENTAÇÃO E NÃO UMA OBRIGAÇÃO, CASO QUEIRA INICIAR UM NOVO TRATAMENTO DURANTE O ESTUDO VOCÊ NÃO SERÁ IMPEDIDO. ALÉM DISSO, VOCÊ NÃO SERÁ SOLICITADO PARAR OS TRTAMENTOS QUE JÁ ESTA FAZENDO. CASO VOCÊ PERCEBA QUE ESTA PIORANDO MUITO DEVE ENTRAR EM CONTATOS CONOSCO PELOS NÚMEROS PRESENTES NESTE DOCUMENTO, PARA QUE POSSAMOS FAZER UMA AVALIAÇÃO EM VOCÊ PARA SABER O QUE ESTA CAUSANDO SUA PIORA. DESSA FORMA CONSIGREMOS TE OFERECER UM TRATAMENTO PARA CORRIGIR SUA PIORA, COMO POR EXEMPLO BOLSA DE ÁGUA QUENTE E ALONGAMENTO LEVE ESCALA PARA DIMINUIR A DOR.”

# Considerações Finais a critério do CEP:

- Segundo a Carta Circular nº. 003/2011/CONEP/CNS, de 21/03/11, no momento da obtenção do TCLE, há obrigatoriedade de rubrica em todas as páginas do mesmo, pelo sujeito de pesquisa ou seu responsável e pelo pesquisador. O pesquisador responsável deverá apor sua assinatura na última página do referido termo.
- Os Relatórios parciais deverão ser apresentados ao CEP em 01/08/2021, 01/02/2022, 01/08/2022, 01/02/2023, 01/08/2023.
- O Relatório final deverá ser apresentado ao CEP ao término do estudo em 01/03/2024. Considera-se como antiética a pesquisa descontinuada sem justificativa aceita pelo CEP que a aprovou.
- Caso haja quaisquer intercorrências durante a execução do projeto de pesquisa é de responsabilidade do pesquisador responsável comunicá-la através de uma emenda ao CEP via Plataforma Brasil. Considera-se como antiética a pesquisa com modificações em seu protocolo inicial previamente aprovado sem justificativa aceita pelo CEP que a aprovou.

O projeto atende aos preceitos éticos para pesquisas envolvendo seres humanos preconizados na Resolução 466/12 CNS.

# Este parecer foi elaborado baseado nos documentos abaixo relacionados:

| Tipo Documento | Arquivo | Postagem | Autor | Situação |
| --- | --- | --- | --- | --- |
| Informações Básicas | PB_INFORMAÇÕES_BÁSICAS_DO_P | 18/10/2020 |  | Aceito |
| do Projeto | ROJETO_1563109.pdf | 15:09:48 |  |  |
| Solicitação | CARTA_RESPOSTA_AS_PENDENCIA | 18/10/2020 | VINICIUS CUNHA | Aceito |
| registrada pelo CEP | S_MODIFICADO.pdf | 15:08:40 | DE OLIVEIRA |  |
| TCLE / Termos de | Termo_de_Consentimento_Livre_e_Escl | 18/10/2020 | VINICIUS CUNHA | Aceito |
| Assentimento / | arecido_Modificado_2.pdf | 14:51:09 | DE OLIVEIRA |  |
| Justificativa de |  |  |  |  |
| Ausência |  |  |  |  |
| Projeto Detalhado / | Projeto_Modificado.pdf | 18/10/2020 | VINICIUS CUNHA | Aceito |
| Brochura |  | 14:50:19 | DE OLIVEIRA |  |
| Investigador |  |  |  |  |
| Projeto Detalhado / | Projeto_Modificado_2.pdf | 18/10/2020 | VINICIUS CUNHA | Aceito |
| Brochura |  | 14:48:21 | DE OLIVEIRA |  |
| Investigador |  |  |  |  |
| Declaração de | Carta_de_coparticipe.pdf | 24/09/2020 | VINICIUS CUNHA | Aceito |
| Instituição e |  | 13:07:52 | DE OLIVEIRA |  |
| Infraestrutura |  |  |  |  |
| TCLE / Termos de | Termo_de_Consentimento_Livre_e_Escl | 24/09/2020 | VINICIUS CUNHA | Aceito |
| Assentimento / | arecido_Modificado.pdf | 12:39:53 | DE OLIVEIRA |  |
| Justificativa de |  |  |  |  |

| Ausência | Termo_de_Consentimento_Livre_e_Escl | 24/09/2020 | VINICIUS CUNHA | Aceito |
| --- | --- | --- | --- | --- |
|  | arecido_Modificado.pdf | 12:39:53 | DE OLIVEIRA |  |
| Folha de Rosto | Folha_de_Rosto_Assinado.pdf | 28/08/2020 | VINICIUS CUNHA | Aceito |
|  |  | 12:14:43 | DE OLIVEIRA |  |
| Projeto Detalhado / | Projeto.pdf | 28/08/2020 | VINICIUS CUNHA | Aceito |
| Brochura |  | 12:11:09 | DE OLIVEIRA |  |
| Investigador |  |  |  |  |
| Orçamento | ORCAMENTO.pdf | 22/07/2020 | VINICIUS CUNHA | Aceito |
|  |  | 23:07:59 | DE OLIVEIRA |  |
| Declaração de | Carta_de_anuencia_Senador_Modestin | 22/07/2020 | VINICIUS CUNHA | Aceito |
| Instituição e | o.pdf | 23:07:26 | DE OLIVEIRA |  |
| Infraestrutura |  |  |  |  |
| Declaração de | CARTA_DE_ANUENCIA.pdf | 22/07/2020 | VINICIUS CUNHA | Aceito |
| Instituição e |  | 23:07:05 | DE OLIVEIRA |  |
| Infraestrutura |  |  |  |  |
| Cronograma | CRONOGRAMA_DE_EXECUAOO.pdf | 22/07/2020 | VINICIUS CUNHA | Aceito |
|  |  | 23:06:28 | DE OLIVEIRA |  |

**Situação do Parecer:**

Aprovado

# Necessita Apreciação da CONEP:

Não

DIAMANTINA, 20 de Outubro de 2020

# Assinado por:

**Raquel Schwenck de Mello Vianna Soares (Coordenador(a))**
