# Supplementary material for: The ESCAPE trial for older people with chronic low back pain: Protocol of a randomized controlled trial
Source: PLoS One. 2022 May 26;17(5):e0266613. doi: 10.1371/journal.pone.0266613 (PMC9135264; doi:10.1371/journal.pone.0266613)
Supplement: S2 File — (DOCX) [file pone.0266613.s005.docx]

**CONSTITUTED OPINION OF A CEP**

# RESEARCH PROJECT DATA

**Research Title: Efficacy of Exercise on Chronic Low Back Pain Pain and Disability in the Elderly: A Randomized Clinical Trial**

**Researcher:** VINICIUS CUNHA DE OLIVEIRA

# Subject Area:

**Version:** 3

**CAAE:** 37088920.5.0000.5108

**Proposing Institution:** Universidade Federal dos Vales do Jequitinhonha e Mucuri

**Main Sponsor:** Financiamento Próprio

# OPINION DATA

**Opinion Number:** 4.350.616

# Project presentation:

“The information listed here was taken from the Basic Research Information file (PB_Informações_Básicas_do_projeto_1563109.pdf, de 18/10/2020).

Low back pain is one of the most common health conditions worldwide. It is estimated that 70% to 85% of the general population has had or will have an episode of low back pain during their lifetime, including the elderly. The most common low back pain is nonspecific, defined as symptoms without a specific cause. High-quality evidence supports active strategies such as exercise for the population to treat general nonspecific low back pain. A previous review by our research group also showed promising efficacy of exercise on low back pain-related pain and disability in the elderly. However, the management of nonspecific low back pain in the elderly population has been neglected in the scientific literature, so the evidence is weak and limited to a small number of randomized controlled trials of low power and high risk of bias. Furthermore, the effectiveness of group exercise has not been investigated in elderly people with non-specific low back pain. OBJECTIVE - To investigate the effect of an 8-week group exercise protocol on pain and disability in elderly people with nonspecific low back pain. METHODS – Elderly people aged 60 years and over will be evaluated in the pre- and post-intervention period, in addition to a 6 and 12-month follow-up. The questionnaires that will be applied are the following: Numerical Pain Scale (0 - 10); Roland-Morris Questionnaire (0 - 24); Global Effect Perception Scale (-5 - 5); Falls

Efficacy Scale – International (16-64); Physical Activity Rating (0 -7). After the pre-intervention evaluation, participants will be randomly distributed between two groups. Control group/Group exercise group. Group exercises will be performed 3 times a week for 8 weeks and the control group will remain on the waiting list. The allocation of participants to each group will be carried out using an opaque envelope.

- Null hypothesis – The group exercise protocol is not different from the control group for pain and disability in elderly people with low back pain.
- Alternative hypothesis – The group exercise protocol improves pain and disability in elderly people with low back pain. These effects are maintained after 6 and 12 months of follow-up. In addition, elderly people who participated in the group will present a higher level of physical activity after 12 months of follow-up.

Inclusion Criteria:

·Elderly people of both sexes aged 60 years or more;

· Complaint of non-specific low back pain lasting at least 3 months;

·Disability with RMDQ score 4/24 or more in the past six months and persistent back pain with pain 3/10 or more in the past six months (RUNDELL et al., 2017).

Exclusion Criteria:

- - Suspected or confirmed severe spinal pathology (fracture, metastatic, inflammatory or infectious diseases of the spine, cauda equina syndrome / generalized neurological disorder);

-Compromise of the nerve root;

-Surgery of the spine;

-Major surgeries scheduled during the treatment or follow-up period;

-Any of the contraindications to exercise listed on page 103 of the ACSM guidelines (DALLECK; TISCHENDORF, 2012) (Annex 1)

# Research Objective:

"The information listed here were taken from the Basic Research Information file (PB_Informações_Básicas_do_projeto_1563109.pdf, of 10/18/2020).

# Primary Objective:

To investigate the effect of an 8-week group exercise protocol on pain and disability in elderly people with non-specific low back pain

Continuação do Parecer: 4.350.616

Secondary Objectives:

- To investigate the effect of an 8-week group exercise protocol on the global impression of recovery in elderly people with non-specific low back pain.
- To investigate the effect of an 8-week group exercise protocol on the fear of falling in elderly people with nonspecific low back pain.
- To investigate the effect of an 8-week group exercise protocol on the frequency of falls in elderly people with nonspecific low back pain.
- To investigate the effect of an 8-week group exercise protocol on the level of physical activity in elderly people with nonspecific low back pain after 12 months of follow-up.

# Risk and Benefits Assessment:

“The information listed here was taken from the Basic Research Information file (PB_Informações_Básicas_do_projeto_1563109.pdf, de 18/10/2020).

Scratchs:

The risks for participating in this study are minimal. INTERVENTION GROUP PARTICIPANTS may experience low intensity muscle pain after performing the exercise program. If it occurs, to minimize pain, thermotherapy will be performed by the researcher and stretching will be advised on a light scale. DUE TO THE FACT THAT THE CONTROL GROUP REMAINS ON THE WAITING LIST (NOT PERFORMING ANY INTERVENTION), THE RISKS ARE EVEN LESS. A POTENTIAL RISK TO PARTICIPANTS OF THIS GROUP IS THE FACT THAT THEY WILL BE ADVICED NOT TO START ANY TREATMENT DURING THE STUDY PROTOCOL, THUS, IT WILL SPEND 8 WEEKS WITHOUT A NEW INTERVENTION TO TREAT YOUR DYSFUNCTION. AS A WAY OF MINIMIZING, PARTICIPANTS WILL BE INFORMED THAT THIS IS AN ORIENTATION, IF THEY WANT TO START A NEW TREATMENT DURING THE STUDY, THEY WILL NOT BE PREVENTED. IN ADDITION, THE INTERRUPTION OF TREATMENTS ALREADY PERFORMED WILL NOT BE REQUESTED. PARTICIPANTS WILL ALSO BE DIRECTED TO CONTACT RESEARCHERS IN CASE OF WORSE IN THE CLINICAL FRAMEWORK, IF IT OCCURS, AN EVALUATION WILL BE CARRIED OUT TO IDENTIFY POSSIBLE CAUSES AND THE PARTICIPANT WILL BE PROVIDED WITH INTERVENTIONS FOR CORRECTION OF THE CLINICAL FRAMEWORK, FOR EXAMPLE ANALGESIA.

Benefits:

Participation in this study will bring direct and indirect benefits. The direct benefits will be through improvements in different health domains caused by physical activity. As indirect benefits, the results of this research may contribute to help the physiotherapy professional in the implementation of effective and financially viable treatment protocols for elderly patients with low back pain.

# Research Comments and Considerations:

“The information listed here was taken from the Basic Research Information file (PB_Informações_Básicas_do_projeto_1563109.pdf, de 18/10/2020).

Data analysis methodology:

Sample size calculation: Sample size calculation was performed using the G*Power software

3.1. The difference between the means of a meta-analysis carried out by our research group (data to be published) was divided by the standard deviation of the study by ZADRO (2019) to obtain Cohen's d. Cohen's d data were used to generate the effect size f. 120 participants are needed (60 in each group), considering a statistical power of 80%, alpha 5% and a dropout rate of 20%.

Analysis of treatment effects.

Statistical analysis will be carried out following the principles of intention-to-treat analysis through the multiple impotation method (MCCOY, 2017). First, the normality of the data will be tested by the Kolmogorov-Smirnov test and the homoscedasticity of the data will be tested by the Levene test. Then, considering the normal distribution, repeated measures multivariate analysis of variance (MANOVA) will be used to determine the effects of 12 weeks of intervention and 6 and 12 months of follow-up. Significant differences detected in the analysis of variance will be examined again with a post hoc LSD analysis with the corrected alpha level of 0.05 for multiple comparisons. The effect size will be calculated and set to small (0.2), medium (0.5) and large (0.8). (COHEN, [s.d.]) The primary outcomes will be analyzed based on the clinically important measure for the elderly population that will be developed during the study. All statistical analyzes will be performed with SPSS version 22, and the results will be presented as means and 95% confidence intervals (CIs). The sample will be dichotomized for improvement/maintenance according to clinically important change for adults.

The absolute risk reduction will be obtained by subtracting the IG risk by the CG risk. These data will be used to calculate the number needed to treat (NNT) (100% / reducing absolute risk). We will dichotomize our sample into two groups based on GPE scores. It will be considered as improvement when participants score 3 (completely recovered) or 2 (very recovered) and stable when they score 1 (slightly recovered) and 0 (no change) or -1 (slightly peep). To determine the clinically important change (MCI) of the QIRM and END we will use a Receiver Operating Characteristic (ROC) curve (DE VET et al., 2007) (PORTNEY; WATKINS, 2008). The cutoff point of the ROC curve will be calculated by identifying the point on the curve closest to the upper left corner, considered the best cutoff for which the sum of the percentages of false positive and false negative classifications ([1 - sensitivity] + [1 - specificity]) is the lowest (DE VET et al., 2007). Responsiveness will be assessed by examining areas under the ROC curve (AUC) and correlations between QIRM and END and GPE change scores. The AUC will be obtained to describe the ability of the QIRM and the END to distinguish participants who showed improvement from stable participants (PORTNEY; WATKINS, 2008). AUC of 0.50 indicates that the questionnaire has no diagnostic accuracy beyond chance, while a value of 1.00 indicates perfect accuracy (PORTNEY; WATKINS, 2008). AUC of at least 0.70 was considered adequate (TERWEE et al., 2007).

Previsão de início do trabalho: 01/02/2021 Work completion forecast: 01/02/2024

# Considerations for Mandatory Submission Terms:

See field: "Conclusions and Pending Issues and List of Inadequacies”

# Recommendations:

See field: "Conclusions and Pending Issues and List of Inadequacies”

# Conclusions or Pending Issues and List of Inadequacies:

“The information listed here was taken from the Basic Research Information file (PB_Informações_Básicas_do_projeto_1563109.pdf, of 10/18/2020).

Pending 1: “Risks and Benefits - does not include the Control Group's risks nor what measures will be taken to minimize them. The volunteer, even in the control group, is the responsibility of the researcher, as he is part of the research.”

Pending Response 1: The topic “Risks and Benefits” was changed to meet the request, thus the following information was inserted: The risks for participating in this study are minimal.

INTERVENTION GROUP PARTICIPANTS may experience low intensity muscle pain after performing the exercise program. If it occurs, to minimize pain, thermotherapy will be performed by the researcher and stretching will be advised on a light scale. DUE TO THE FACT THAT THE CONTROL GROUP REMAINS ON THE WAITING LIST (NOT PERFORMING ANY INTERVENTION), THE RISKS ARE EVEN LESS. A POTENTIAL RISK TO PARTICIPANTS OF THIS GROUP IS THE FACT THAT THEY WILL BE ADVICED NOT TO START ANY TREATMENT DURING THE STUDY PROTOCOL, THUS, IT WILL SPEND 8 WEEKS WITHOUT A NEW INTERVENTION TO TREAT YOUR DYSFUNCTION. AS A WAY OF MINIMIZING, PARTICIPANTS WILL BE INFORMED THAT THIS IS AN ORIENTATION, IF THEY WANT TO START A NEW TREATMENT DURING THE STUDY, THEY WILL NOT BE PREVENTED. IN ADDITION, THE INTERRUPTION OF TREATMENTS ALREADY PERFORMED WILL NOT BE REQUESTED. PARTICIPANTS WILL ALSO BE DIRECTED TO CONTACT RESEARCHERS IN CASE OF WORSE IN THE CLINICAL FRAMEWORK, IF IT OCCURS, AN EVALUATION WILL BE CARRIED OUT TO IDENTIFY POSSIBLE CAUSES AND THE PARTICIPANT WILL BE PROVIDED WITH INTERVENTIONS FOR CORRECTION OF THE CLINICAL FRAMEWORK, FOR EXAMPLE ANALGESIA.

Pending 2: "Co-participant letter from Senator Modestino Gonçalves - this should be reworked, as

it is not a letter of consent, but a Co-participating Institution. This should be dated after this initial review of this CEP. So it can already be added in the new submission.”

Pending Response 2: Senator Modestino Gonçalves' co-participant letter was inserted as an attachment on the platform to this submission.

Pending 3 “TCLE - it is necessary to describe which exercises will be performed by the individuals in the intervention group (participants need to have information about the exercises performed). In addition, as mentioned above, it is necessary to describe the risks of the control group and how to minimize them.

Pending response 3: The descriptions of the exercises were inserted in the TCLE as requested as follows: “THE EXERCISES THAT WILL BE PERFORMED ARE OFTEN USED IN GROUP EXERCISE PROGRAMS AND WILL BE THE FOLLOWING:

1- STRENGTHENING OF THE LEGS - EXERCISES WILL BE PERFORMED SUCH AS SITTING AND RISING FROM A CHAIR, SQUATTING WITH ONE LEG IN FRONT OF THE OTHER, STAYING ON TIPS, OPENING AND CLOSING THE LEGS LYING ON THE SIDE, RAISING AND LOWERING THE LEG LYING TO THE BELLY DOWN, ASCENDING AND DESCENDING STEPS.

1. BODY STRENGTHENING - EXERCISES WILL BE PERFORMED, SUCH AS BRIDGE EXERCISE, BOARD KEEPING THE BODY STRAIGHT ON THE ELBOW AND FEET, ABDOMINAL STRENGTHENING, BICYCLE MOVEMENT BICYCLE SITTING UP
2. STRENGTHENING OF THE ARMS – EXERCISES WILL BE PERFORMED, SUCH AS RAISING THE ARMS TO THE HEIGHT OF THE SHOULDERS, MAKES THE BENDING MOVEMENT ON THE WALL AND A BENDING MOVEMENT ON THE KNEE.
3. BALANCE EXERCISES SUCH AS STANDING ON ONE LEG, WALK WITH THE HEEL OF ONE FOOT ON THE TIP OF THE OTHER, DODGE CONES OF CONES, PASS OVER SHOE BOXES.
4. 10-MINUTE WALK.”

The risks for the control group were described in the informed consent form as follows:

“IF YOU ARE DRAWNED TO THE CONTROL GROUP, DUE TO THE FACT OF REMAIN ON THE WAITING LIST (NOT PERFORMING ANY TREATMENT) THE RISKS ARE EVEN LESS. A RISK THAT MAY BE MENTIONED IS THE FACT THAT YOU WILL BE ADVICED NOT TO START ANY TREATMENT DURING THE STUDY PROTOCOL, THUS YOU WILL SPEND 8 WEEKS WITHOUT A NEW TREATMENT FOR YOUR BACK PAIN. AS A WAY TO MINIMIZE THIS RISK, WE MAKE IT CLEAR THAT THIS IS AN OBLIGATION AND NOT AN OBLIGATION, IF YOU WANT TO START A NEW TREATMENT DURING THE STUDY YOU WILL NOT BE STOPPED. IN ADDITION, YOU WILL NOT BE REQUESTED TO STOP THE TREATMENTS YOU ARE ALREADY MAKING. IF YOU UNDERSTAND THAT THIS IS WORSE A LOT, YOU SHOULD CONTACT US BY THE NUMBERS IN THIS DOCUMENT, SO THAT WE CAN MAKE AN EVALUATION ON YOU TO KNOW WHAT IS CAUSING YOUR WORSE. IN THIS WAY WE WILL BE ABLE TO OFFER YOU A TREATMENT TO CORRECT YOUR WORSE, SUCH AS A HOT WATER BAG AND LIGHT STRETCHING SCALE TO REDUCE THE PAIN.”

# Final Considerations at the discretion of the CEP:

According to Circular Letter no. 003/2011/CONEP/CNS, of 03/21/11, at the time of obtaining the TCLE, there is a mandatory rubric on all its pages, by the research subject or his/her responsible person and by the researcher. The responsible researcher must put his/her signature on the last page of the referred term.

- Partial Reports must be submitted to the CEP on 08/01/2021, 02/01/2022, 08/01/2022, 02/01/2023, 08/01/2023.

- The final report must be presented to the CEP at the end of the study on 03/01/2024. Discontinued research without justification accepted by the CEP that approved it is considered unethical.
- f there are any complications during the execution of the research project, it is the responsibility of the responsible researcher to communicate them through an amendment to the CEP via Plataforma Brasil. Research with modifications to its previously approved initial protocol without justification accepted by the CEP that approved it is considered unethical.

The project meets the ethical precepts for research involving human beings recommended in Resolution 466/12 CNS.

# This opinion was prepared based on the documents listed below:

| Document Type | Archive | Post | Auth  or | Situation |
| --- | --- | --- | --- | --- |
| Basic information | PB_INFORMAÇÕES_BÁSICAS_DO_P | 18/10/2020 |  | Accepted |
| of project | ROJETO_1563109.pdf | 15:09:48 |  |  |
| Solicitation | CARTA_RESPOSTA_AS_PENDENCIA | 18/10/2020 | VINICIUS CUNHA | Accepted |
| registered by the CPE | S_MODIFICADO.pdf | 15:08:40 | DE OLIVEIRA |  |
| TCLE / Terms of | Termo_de_Consentimento_Livre_e_Escl | 18/10/2020 | VINICIUS CUNHA | Accepted |
| Assent / | arecido_Modificado_2.pdf | 14:51:09 | DE OLIVEIRA |  |
| Justification of |  |  |  |  |
| Absence |  |  |  |  |
| Detailed project / | Projeto_Modificado.pdf | 18/10/2020 | VINICIUS CUNHA | Accepted |
| Brochure |  | 14:50:19 | DE OLIVEIRA |  |
| Investigator |  |  |  |  |
| Detailed project / | Projeto_Modificado_2.pdf | 18/10/2020 | VINICIUS CUNHA | Accepted |
| Brochure |  | 14:48:21 | DE OLIVEIRA |  |
| Investigator |  |  |  |  |
| Declaration of | Carta_de_coparticipe.pdf | 24/09/2020 | VINICIUS CUNHA | Accepted |
| Institution and |  | 13:07:52 | DE OLIVEIRA |  |
| Infrastructure |  |  |  |  |
| TCLE / Terms of | Termo_de_Consentimento_Livre_e_Escl | 24/09/2020 | VINICIUS CUNHA | Accepted |
| Assent / | arecido_Modificado.pdf | 12:39:53 | DE OLIVEIRA |  |
| Justification of |  |  |  |  |

| Absence | Termo_de_Consentimento_Livre_e_Escl | 24/09/2020 | VINICIUS CUNHA | Accepted |
| --- | --- | --- | --- | --- |
|  | arecido_Modificado.pdf | 12:39:53 | DE OLIVEIRA |  |
| cover sheet | Folha_de_Rosto_Assinado.pdf | 28/08/2020 | VINICIUS CUNHA | Accepted |
|  |  | 12:14:43 | DE OLIVEIRA |  |
| Detailed project / | Projeto.pdf | 28/08/2020 | VINICIUS CUNHA | Accepted |
| Brochure |  | 12:11:09 | DE OLIVEIRA |  |
| Investigator |  |  |  |  |
| Budget | ORCAMENTO.pdf | 22/07/2020 | VINICIUS CUNHA | Accepted |
|  |  | 23:07:59 | DE OLIVEIRA |  |
| Declaration of | Carta_de_anuencia_Senador_Modestin | 22/07/2020 | VINICIUS CUNHA | Accepted |
| Institution and | o.pdf | 23:07:26 | DE OLIVEIRA |  |
| Infrastructure |  |  |  |  |
| Declaration of | CARTA_DE_ANUENCIA.pdf | 22/07/2020 | VINICIUS CUNHA | Accepted |
| Institution and |  | 23:07:05 | DE OLIVEIRA |  |
| Infrastructure |  |  |  |  |
| Schedule | CRONOGRAMA_DE_EXECUAOO.pdf | 22/07/2020 | VINICIUS CUNHA | Accepted |
|  |  | 23:06:28 | DE OLIVEIRA |  |


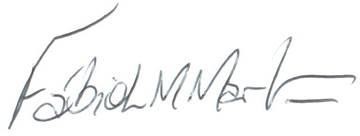
**Status of Opinion:**

Approved

# Needs Appreciation of CONEP:

No

DIAMANTINA, October 20, 2020
